# Genetic profiling of fatty acid desaturase polymorphisms identifies patients who may benefit from high-dose omega-3 fatty acids in cardiac remodeling after acute myocardial infarction—Post-hoc analysis from the OMEGA-REMODEL randomized controlled trial

**DOI:** 10.1371/journal.pone.0222061

**Published:** 2019-09-18

**Authors:** Raymond Y. Kwong, Bobak Heydari, Yin Ge, Shuaib Abdullah, Kana Fujikura, Kyoichi Kaneko, William S. Harris, Michael Jerosch-Herold, Elliott M. Antman, Jonathan G. Seidman, Marc A. Pfeffer

**Affiliations:** 1 Noninvasive Cardiovascular Imaging Section, Cardiovascular Division, Department of Medicine and Department of Radiology, Brigham and Women’s Hospital, Boston, Massachusetts, United States of America; 2 Cardiovascular Division, Department of Medicine, Brigham and Women’s Hospital, Boston, Massachusetts, United States of America; 3 Cardiovascular Division, Department of Medicine, University of Calgary, Calgary, Alberta, Canada; 4 Department of Internal Medicine, Sanford School of Medicine, University of South Dakota, Sioux Fall, South Dakota, United States of America; 5 OmegaQuant Analytics, LLC, Sioux Falls, South Dakota, United States of America; 6 Department of Genetics, Harvard Medical School, Boston, Massachusetts, United States of America; University of Messina, ITALY

## Abstract

**Background:**

The double-blind OMEGA-REMODEL placebo-controlled randomized trial of high-dose omega-3 fatty acids (O-3FA) post-acute myocardial infarction (AMI) reported improved cardiac remodeling and attenuation of non-infarct myocardial fibrosis. Fatty acid desaturase 2 (FADS2) gene cluster encodes key enzymes in the conversion of essential omega-3 and omega-6 fatty acids into active arachidonic (ArA) and eicosapentaenoic acids (EPA), which influence cardiovascular outcomes.

**Methods and results:**

We tested the hypothesis that the genotypic status of FADS2 (rs1535) modifies therapeutic response of O-3FA in post-AMI cardiac remodeling in 312 patients. Consistent with known genetic polymorphism of FADS2, patients in our cohort with the guanine-guanine (GG) genotype had the lowest FADS2 activity assessed by arachidonic acid/linoleic acid (ArA/LA) ratio, compared with patients with the adenine-adenine (AA) and adenine-guanine (AG) genotypes (GG:1.62±0.35 vs. AA: 2.01±0.36, p<0.0001; vs. AG: 1.76±0.35, p = 0.03). When randomized to 6-months of O-3FA treatment, GG patients demonstrated significant lowering of LV end-systolic volume index (LVESVi), N-terminal prohormone of brain natriuretic peptide (NT-proBNP), and galectin-3 levels compared to placebo (-4.4 vs. 1.2 ml/m^2^, -733 vs. -181 pg/mL, and -2.0 vs. 0.5 ng/mL; p = 0.006, 0.006, and 0.03, respectively). In contrast, patients with either AA or AG genotype did not demonstrate significant lowering of LVESVi, NT-proBNP, or galectin-3 levels from O-3FA treatment, compared to placebo. The odds ratios for improving LVESVi by 10% with O-3FA treatment was 7.2, 1.6, and 1.2 in patients with GG, AG, and AA genotypes, respectively.

**Conclusion:**

Genetic profiling using FADS2 genotype can predict the therapeutic benefits of O-3FA treatment against adverse cardiac remodeling during the convalescent phase of AMI.

**Clinical trial registration information:**

clinicaltrials.gov Identifier: NCT00729430.

## Introduction

Despite significant advances in the treatment of acute myocardial infarction (AMI), adverse left ventricular (LV) remodeling remains prevalent and is a key risk factor for cardiovascular (CV) death, ventricular arrhythmias, and progression to heart failure [1]. In the randomized, placebo-controlled Effect of Omega-3 Acid Ethyl Esters on Left Ventricular Remodeling After Acute Myocardial Infarction (OMEGA-REMODEL) trial [2], our group observed significant attenuation of adverse LV remodeling and non-infarct myocardial fibrosis following AMI with 6-months of high-dose omega-3 fatty acids (O-3FA, 4 grams/day), in addition to guideline-based invasive and medical therapies.

Essential fatty acids, linoleic acid (LA, 18:2n-6) and alpha-linolenic acid (ALA, 18:3n-3), are metabolized and elongated into long-chain polyunsaturated fatty acids through the actions of a series of fatty acid desaturases. A key enzyme in this pathway, delta-6 desaturase encoded by the fatty acid desaturase 2 gene (FADS2, rs1535), converts LA and ALA into arachidonic acid (ArA, 20:4n-6) and eicosapentaenoic acids (EPA, 20:5n-3), respectively. Patients with the guanine-guanine (GG) genotype of this single nucleotide polymorphism of FADS2 (rs1535) have demonstrated increased risk of inflammatory conditions and therapeutic benefit from O-3FA [3–5]. Maladaptive inflammation following AMI plays an important role in preventing adequate tissue healing with consequent increased myocardial fibrosis and biomechanical strain, which promotes adverse left ventricular remodeling and contributes to a state of persistent inflammation that can result in a negative feedback loop [6, 7]. Recently discovered bioactive mediators enzymatically produced from polyunsaturated fatty acids have been shown to actively resolve inflammation and promote removal of dead cells involved in tissue repair [8]. Myocardial recovery post infarction may be determined by the active transition from initial proinflammatory promoters to specialized proresolving lipid mediators (SPMs). O-3FAs are a rich source of SPMs [9, 10] but are poorly consumed in western diets [11]. In this post-hoc analysis of the OMEGA-REMODEL trial, we hypothesized that personalized profiling of FADS2 genetic polymorphisms would result in an improved selection of patients who would respond favorably to O-3FA therapy by attenuating adverse left ventricular functional deterioration and non-infarct myocardial fibrosis during the convalescent healing phase following AMI.

## Materials and methods

### The OMEGA-REMODEL randomized controlled trial

The main results of the OMEGA-REMODEL trial have been published [2]. OMEGA-REMODEL was a prospective, double-blind, placebo-controlled trial of 358 patients randomized to 4 grams/day of O-3FA for 6-months following an acute MI (2–4 weeks). Patient subjects were enrolled from 3 tertiary-care centers in Boston, Massachusetts. Inclusion criteria included all adult patients with symptoms of an acute coronary syndrome, serum troponin elevation consistent with acute myocardial injury, and angiographically significant obstructive coronary artery disease. Exclusion criteria included non-cardiac comorbidities with <1-year life expectancy, active pregnancy, and any absolute contraindications to contrast-enhanced cardiac magnetic resonance imaging (CMR). Patients self-reported their racial and ethnic categories as Caucasian, Black, Hispanic, Asian, Native Hawaiian or Pacific Islander, or other. The trial protocol included baseline CMR, red blood cell omega fatty acids quantitation, and plasma biomarker profile upon study entry and repeated after 6-month therapy with O-3FA or placebo. Primary study endpoint was adverse LV remodeling measured as change in left ventricular end-systolic volume indexed to body surface area (LVESVi, mL/m^2^) by CMR after 6 months. Secondary endpoints included changes in non-infarct myocardial fibrosis measured as the myocardial extracellular volume fraction (ECV) remote from the acute infarction, infarct size, and left ventricular ejection fraction (LVEF). The study protocol was approved by the institutional review board of each enrolling site (Brigham and Women's Hospital, Massachusetts General Hospital, Beth Israel Deaconess Medical Center). All patients provided written informed consent.

### Omega fatty acids levels

Blood samples were collected at both baseline and 6-month follow-up study visits immediately prior to CMR studies. Plasma and serum were isolated within 45 minutes of collection, frozen at -80 degrees Celsius, and were batch shipped to Health Diagnostic Laboratory, Inc. (Richmond, Virginia), for biomarker processing [2]. DNA isolation was obtained from the white blood cell layer after centrifugation. Red blood cell (RBC) fatty acid levels were evaluated using gas chromatography by flame ionization detection (OmegaQuant Analytics, LLC, Sioux Falls, SD). The omega-3 index (O3I) was calculated from the sum of EPA and docosahexaenoic acid (DHA, 20:6n-3) and expressed as a percentage of total RBC fatty acids. Serum biomarkers relating to systemic inflammation (high sensitivity C-reactive protein, hsCRP), innate immunology (myeloperoxidase), lipids, and myocardial stretch or strains (NT-proBNP and Galectin-3) were collected at baseline and after 6 months of study treatment.

### Assessment of rs1535 genetic variant and FADS2 activity

The FADS2 enzyme is a rate-limiting enzyme in the synthesis of long-chain-purified unsaturated fatty acids. We investigated the genetic variant of the FADS2 gene cluster, rs1535 single-nucleotide polymorphism. Isolated DNA underwent genotyping for single-nucleotide polymorphism (SNP) at rs1535 to evaluate genes encoding FADS2 enzymes using Infinium Omni2.5 microarray (Illumina, San Diego, California). The minor guanine (G) allele of rs1535 is associated with reduced desaturation and resultant accumulation of the substrates LA and ALA, and decreased production of ArA, EPA and docosahexaenoic acid (DHA, 22:6n-3). We evaluated FADS2 activity by measured ArA/LA ratio [5].

### Cardiac magnetic resonance

**Fig 1:**
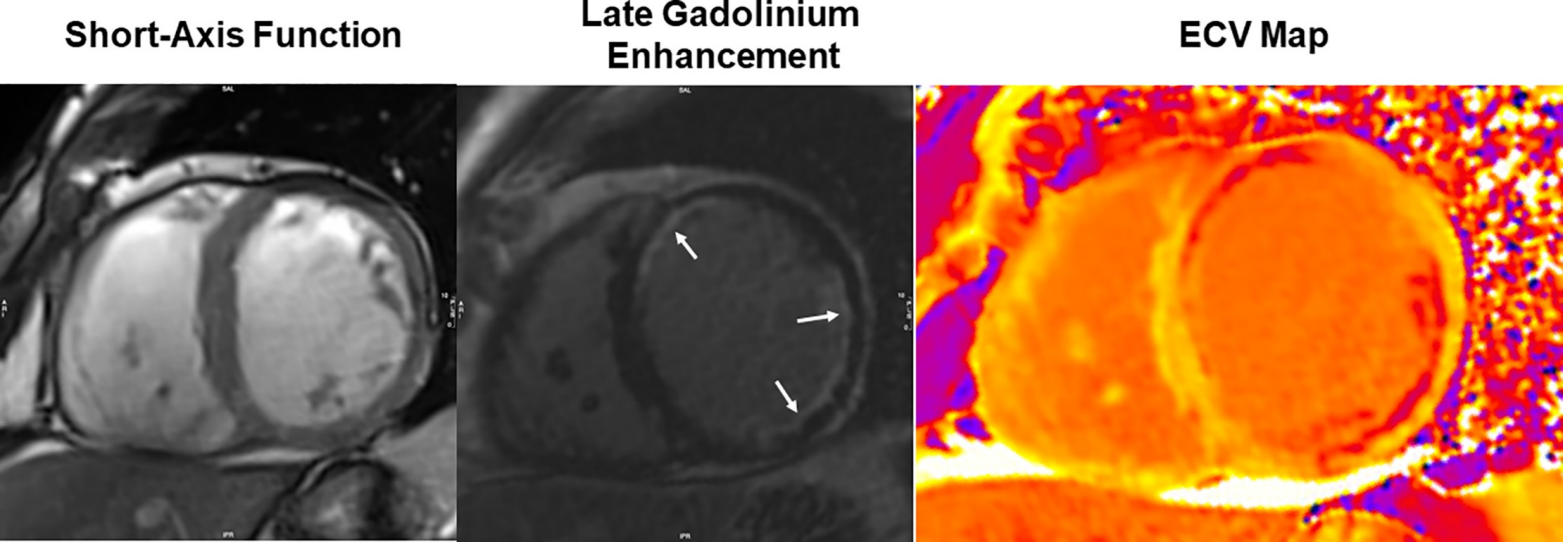
Quantitative assessment of cardiac remodeling and myocardial fibrosis by cardiac magnetic resonance imaging. From left to right, a short-axis cine image, a late gadolinium enhancement (LGE) infarction image, and a myocardial extracellular volume (ECV) map in a matching short-axis location of the left ventricle are shown, respectively. LGE characterizes areas of subendocardial infarct (white arrows). An ECV map was constructed by serial T1 maps before and after gadolinium contrast injection. Non-infarct fibrosis was determined from the ECV maps with matching segments containing LGE removed.

All CMR studies were performed with a 3.0 Tesla scanner (Tim Trio or Verio, Siemens, Erlangen, Germany). The CMR protocol for all patients included assessment of ventricular volumes and function, infarct size and location, and diffuse myocardial fibrosis of non-infarcted myocardium. Infarct size was assessed using late gadolinium enhancement (LGE) imaging, while non-infarct myocardial fibrosis was assessed by quantitation of the ECV using both native and serial post-contrast myocardial T1 mapping (**Fig 1**). Myocardial T1 was measured using a look-locker gradient-echo sequence (3 short-axis locations centered mid-ventricle) acquired prior to and 5, 15, and 25 minutes after administration of 0.1 mmol/kg of intravenous gadolinium (Magnevist, Bracco). Commercial software (QMass®, Medis Inc., Raleigh, North Carolina) was used to analyze images by study personnel blinded to all clinical and biomarker data, time order of the CMR relative to treatment period, and treatment assignment. Total infarct size was measured as infarct mass (in grams) and as percentage of total LV mass from LGE images using a threshold signal intensity ≥2 standard deviations beyond mean of myocardium remote from the infarct [12]. Short-axis LGE and myocardial T1 images were segmented as per the American Heart Association 16-segment model [13]. For each T1 Look-Locker acquisition, T1 was determined by non-linear least squares fitting of a parameterized representation of an inversion recovery (signal intensity = A—B·exp(-TI/T_1_*)) to the measured average signal intensity values in myocardial segments. T1 was then calculated from the best-fit parameters with the correction formula T_1_ = T_1_*·(B/A—1) [14]. ECV was derived by plotting the reciprocal of T1 (R_1_ = 1/T_1_) for myocardial segments against the simultaneously measured R1 in the blood pool, using both pre- and post-contrast measurements where R1 in the blood pool was below 3.5 s^-1^. R1 data pairs with higher values of R1 in the blood pool were excluded from a linear regression line fit to the R1 data to avoid an underestimation of ECV as conditions of fast water-exchange may not be met [15]. ECV was calculated from the slope of the linear regression line, i.e. the partition coefficient (λ), using the blood hematocrit (HCT): ECV = λ·(1-HCT) [16]. ECV segments without matching late enhancement were averaged to yield the global ECV of non-infarcted myocardium (ECV_Non-Infarct_).

### Statistical analysis

Categorical variables were presented as counts with percentages, while continuous variables were expressed as means ± standard deviation. Serum biomarkers were log transformed, where appropriate, to improve statistical power. OM3I was evaluated as a continuous variable, in addition to being divided by median value. Chi-square and ANOVA were used to compare baseline characteristics across FADS2 genotype (AA, AG, GG). Relationships between biomarker levels and CMR variables were evaluated with linear regression analyses. All statistical analyses were performed with SAS (SAS Institutes, version 9.4, Cary, NC), and a p-value < 0.05 was used to ascribe statistical significance.

## Results

### Clinical characteristics by FADS2 genotypes

FADS2 genotyping was conducted in 312 patients and there was no indication of deviation in genotype distribution from Hardy-Weinberg equilibrium (AA:156, 50%; AG:122, 39%; and GG:34, 11%; p = 0.28). Mean age in the overall cohort was 59 ± 10 years and 19% were female. STEMI was the index event in 59% of cases, non-STEMI in the remaining. Baseline demographics stratified by genotype are presented in **Table 1**. Presence of cardiovascular risk factors, location of myocardial infarction, as well as size of infarct measured by peak cardiac-enzyme biomarkers did not differ significantly between genotypes. There were no significant differences in baseline demographics apart from a trend towards higher non-Caucasian race and higher body mass index in the AA group (p = 0.09 and p = 0.008, respectively). Overall, enrolled patients were well-treated, with 93% achieving TIMI 3 flow within the infarct related artery. There was high adherence to post-MI guideline-recommended therapies across all groups, with >90% of patients on all of dual antiplatelet agents, beta-blockers, and statins. Baseline CMR characteristics stratified by FADS2 genotype are shown in **Table 2**. LVEF was preserved at 54 ± 9% overall, while median infarct size was 13 grams (12% of total LV mass). Pre-treatment LVESVi, non-infarct myocardial fibrosis, infarct size by LGE, and RV size and function were not significantly different between the 3 FADS2 genotypes.

**Table 1:** Baseline clinical characteristics stratified by FADS2 genotype (N = 312) of the intention-to-treat study cohort.

Characteristic	FADS2 Genotype				
	AA (n = 156)	AG (n = 122)	GG (n = 34)		P Value *trend*
**Demographics**						
Age—yr	59 ± 11	59 ± 10	59 ± 9		0.91
Female sex–no. (%)	33 (21)	23 (19)	3 (9)		0.14
Caucasian race–no. (%)	121 (78)	99 (81)	31 (91)		0.09
Body mass index [kg/m^2^]	30 ± 6	28 ± 6	27 ± 3		0.008
Heart rate [bpm]	66 ± 12	66 ± 13	67 ± 12		0.92
Systolic BP [mm Hg]	121 ± 14	122 ± 17	120 ± 14		0.91
Diastolic BP [mm Hg]	71 ± 10	70 ± 11	69 ± 8		0.64
**Index Event**						
STEMI–no. (%)	93 (60)	70 (57)	21 (62)		0.99
Anterior MI–no. (%)	40 (26)	39 (32)	10 (29)		0.43
TIMI 3 flow achieved–no. (%)§	124 (91)	110 (95)	32 (97)		0.11
Troponin-T (peak) [μmol/L]*	3.5 (0.9, 16.4)	3.2 (0.9, 10.2)	2.9 (0.7, 8.0)		0.30
Creatine kinase (peak) [U/L]*	736 (313, 1675)	721 (296, 1557)	680 (385, 1797)		0.96
Creatine kinase MB (peak) [U/L]*	77 (27, 161)	54 (21, 146)	61 (16, 138)		0.39
Hematocrit (%)	39 ± 6	40 ± 5	40 ± 4		0.53
**Cardiovascular Disease History**			
Angina–no. (%)	33 (21)	34 (28)	11 (32)		0.10
Prior Myocardial infarction–no. (%)	14 (9)	13 (11)	2 (5)		0.62
CABG–no. (%)	16 (10)	12 (10)	3 (9)		0.78
Peripheral arterial disease–no. (%)	9 (6)	7 (6)	4 (12)		0.35
NYHA class–no. (%)					0.42
1	145 (92)	112 (92)	33 (97)		
2	13 (8)	10 (8)	0 (0)		
3	0 (0)	0 (0)	1 (3)		
Hypercholesterolemia–no. (%)	108 (70)	83 (68)	27 (79)		0.53
Diabetes mellitus–no. (%)	45 (29)	30 (25)	5 (15)		0.08
Hypertension–no. (%)	104 (68)	77 (63)	19 (56)		0.18
Smoker (current)–no. (%)	24 (32)	16 (26)	4 (24)		0.61
**Medications**						
Dual antiplatelet–no. (%)^**γ**^	154 (97)	123 (100)	37 (100)		0.08
Beta-blocker–no. (%)	140 (91)	112 (92)	34 (100)		0.14
Statin–no. (%)	149 (97)	119 (98)	32 (94)		0.68
Calcium channel blocker–no. (%)	15 (10)	8 (7)	1 (3)		0.14
ACE inhibitor or ARB–no. (%)	112 (73)	90 (74)	28 (82)		0.34
Oral hypoglycemic agents–no (%)	25 (16)	24 (20)	3 (9)		0.66
Insulin–no. (%)	19 (12)	9 (7)	1 (3)		0.05
Nitroglycerin–no. (%)	21 (14)	16 (13)	4 (12)		0.78
**Serum Lipid and Biomarker Levels (mg/dL)**			
Total Cholesterol*	129 (107, 149)	129 (109, 151)	126 (106, 142)		0.15
LDL-C*	68 (55, 83)	68 (54, 87)	66 (54, 78)		0.27
HDL-C*	41 (33, 48)	43 (37, 51)	43 (36, 47)		0.58
Triglycerides*	122 (89, 167)	119 (91, 161)	132 (87, 197)		0.13
Serum ST2*	33 (27, 41)	36 (30, 44)	36 (30, 44)		0.58
High Sensitivity CRP*	3 (1.4, 10.6)	2.3 (1.1, 5.6)	1.8 (0.7, 8.3)		0.79
Lipoprotein A*	30 (10, 66)	23 (11, 52)	27 (13, 40)		0.27
Myeloperoxidase*	341 (266, 408)	324 (255, 391)	329 (261, 421)		0.22
Lipoprotein Phospholipase A2*	171 (140, 200)	158 (133, 196)	172 (137, 196)		0.83
Galectin-3*	15 (12, 19)	15 (13, 18)	16 (12, 18)		0.45
NT-proBNP*	446 (236, 881)	479 (233, 934)	508 (136, 1272)		0.24

ACE denotes angiotensin converting enzyme, ARB angiotensin receptor blocker, BP blood pressure, CABG coronary artery bypass grafting, GFR glomerular filtration rate, HDL-C high-density lipoprotein cholesterol, hsCRP high-sensitivity C-reactive protein, LDL-C low-density lipoprotein cholesterol, Lp-PLA2 lipoprotein- associated phospholipase A2, MI myocardial infarction, NT-proBNP N-terminal of the prohormone brain natriuretic peptide, NYHA New York Heart Association, RBC red blood cell, ST2 serum soluble ST2, and STEMI ST elevation myocardial infarction.

*Natural logarithm transformation was used to improve normality and homoscedasticity of residuals.

^§^TIMI describes the thrombolysis in myocardial infarction academic working group.

^**γ**^Dual antiplatelet therapy included aspirin plus either clopidogrel or prasugrel.

**Table 2:** Baseline CMR characteristics stratified by FADS genotype (N = 312) of the intention-to-treat study cohort.

Characteristic	FADS2 Genotype			
	AA (n = 156)	AG (n = 122)	GG (n = 34)	P Value
**Cardiac Magnetic Resonance Imaging**				
LVESVI [mL/m^2^]*	35 (27, 45)	36 (29, 43)	38 (31, 50)	0.33
ECV_Non-Infarct_ [%]¶	34 ± 5	34 ± 5	35 ± 5	0.28
Infarct size [grams using 2SD]*	14 (6, 24)	12 (6, 23)	18 (4, 25)	0.61
Infarct percent (% LV mass)*	12 (6, 22)	10 (5, 20)	16 (4, 24)	0.70
LVEF [%]	54 ± 9	55 ± 9	52 ± 10	0.36
LVEDVI [mL/m^2^]	82 ± 20	84 ± 19	87 ± 22	0.51
RVEF [%]	54 ± 7	53 ± 7	52 ± 5	0.14
RVEDVI [mL/m^2^]	73 ± 19	72 ± 20	70 ± 16	0.69
RVESVI [mL/m^2^]	33 ± 11	34 ± 10	34 ± 9	0.98
LV mass index [g/m^2^]	59 ± 14	62 ± 14	57 ± 13	0.12

LV was defined as left ventricular, LVEDVI left ventricular end-diastolic volume index, LVEF left ventricular ejection fraction, LVESVI left ventricular end-systolic volume index, RVEDVI right ventricular end-diastolic volume index, RVEF right ventricular ejection fraction, and RVESVI right ventricular end-systolic volume index.

Continuous variables are expressed as means ± SD if normally distributed, otherwise median (25^th^, 75^th^ percentile).

^¶^ECV_Remote_ was the extracellular volume fraction of myocardium remote from the infarction, an estimate of non-infarct fibrosis.

*Natural logarithm transformation was used to improve normality and/or homoscedasticity of residuals, prior to performing Student’s t-tests.

### Fatty acid desaturase activity stratified by FADS2 genotype

**Fig 2:**
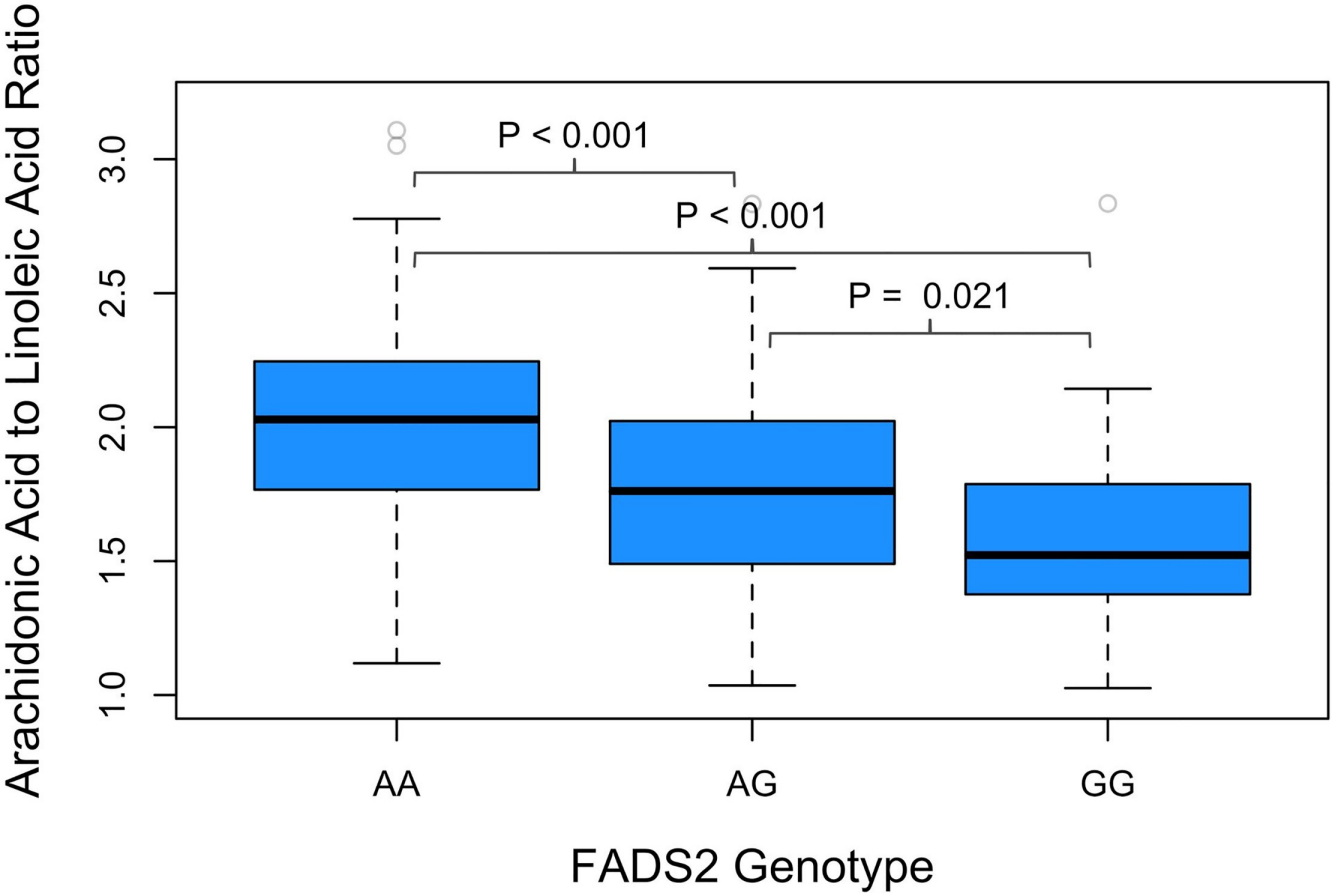
Markedly different fatty acid desaturase activity was seen in patients with various rs-1535 genotypes. Fatty acid desaturase activity was assessed by the ArA/LA ratio in red blood cell membrane assay. Note that patients with GG alleles have diminished ArA/LA ratio consistent with reduced fatty acid desaturase activity.

ArA/LA ratio, as a surrogate measure to estimate fatty acid desaturase activity, demonstrated significant variation between the FADS2 genotypes (**Fig 2**). Patients with the AA genotype had the highest ArA/LA ratio whereas those with the GG genotype had the lowest (AA vs GG, 2.01±0.36 vs 1.62±0.35; p<0.0001). AG patients had an intermediate level of ArA/LA (AA vs AG 2.01±0.36 vs 1.76±0.35; p<0.0001 and AG vs GG 1.76±0.35 vs 1.62±0.35, P = 0.03). This pattern of ArA/LA ratio across the 3 genotypes, was consistent in patients who were randomized to receive O-3FA and placebo.

### Effectiveness of O-3FA in improving post-MI cardiac remodeling across the FADS2 genotypes

**Fig 3:**
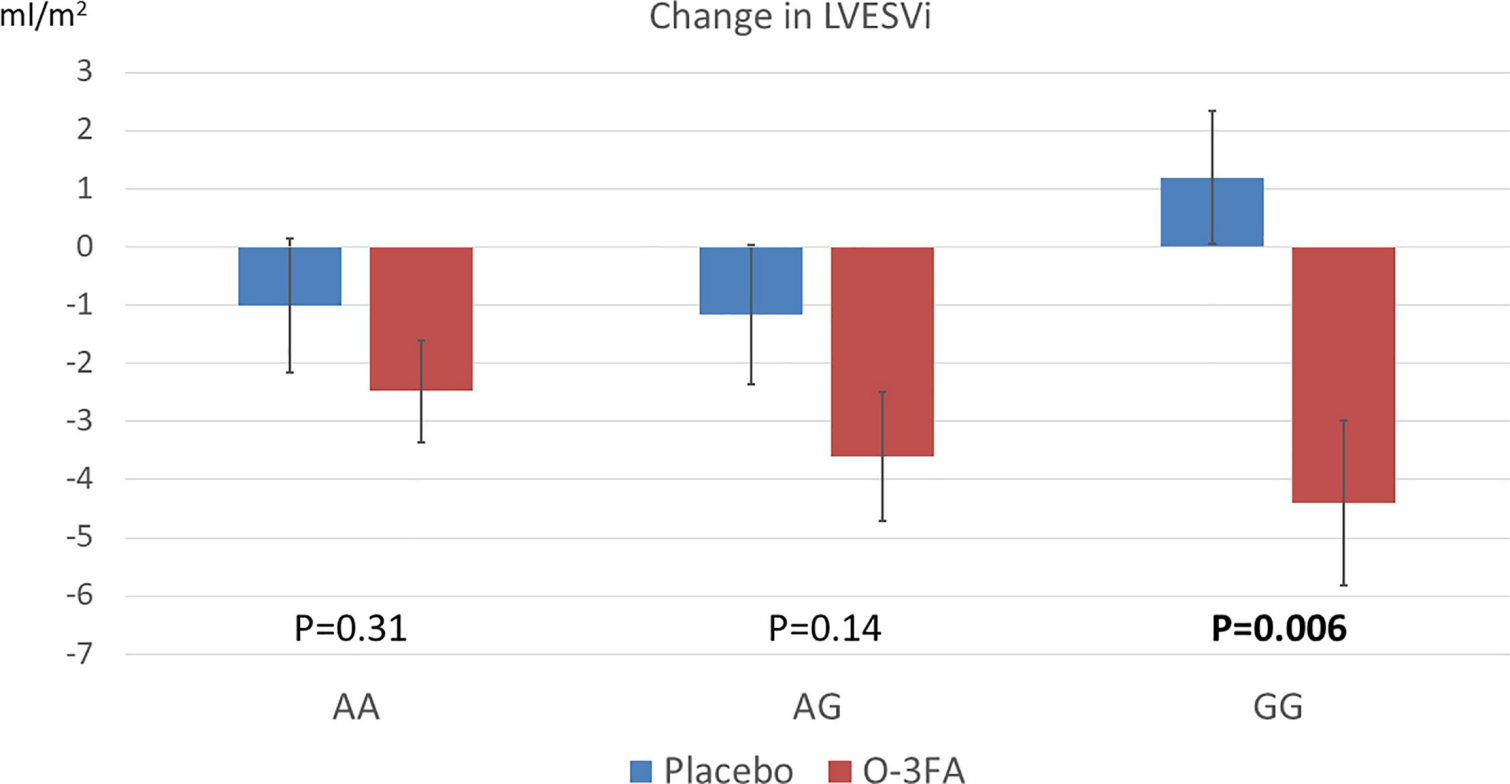
Significant improvement of post-AMI LV remodeling from omega-3 fatty acids treatment in patients with the rs1535 GG genotype. Note the divergent response in LVESVi improvement over the first 6 months after the index AMI in patients with GG allele who were assigned O-3FA compared to placebo. This divergence of cardiac remodeling response to O-3FA treatment was not observed in the AA and AG groups.

In the overall cohort, patients who received O-3FA experienced a mean reduction of LVESVi by 3.1 ml/m^2^, compared to a mean 0.8 ml/m^2^ reduction in the placebo group (P = 0.02). **Fig 3** illustrates the primary endpoints of change in LVESVi, stratified by genotype and treatment assignment. When stratified by genotype, AA and AG patients treated with O-3FA did not experience significant improvement in LVESVi compared to placebo, whereas those who had a GG genotype demonstrated significant improvement of LVESVi when assigned to O-3FA (change in LVESVi -4.4±5.6 vs 1.2±4.1 ml/m^2^, O-3FA vs placebo, p = 0.006). **Table 3** shows changes in CMR parameters stratified by genotype and treatment assignment. The odds ratios for improving LVESVi by 10% with O-3FA treatment were 7.2, 1.6, and 1.2 in patients with GG, AG, and AA genotypes, respectively. Patients with the GG genotype achieved a trend towards LVEDVi reduction when treated with O-3FA compared to placebo, whereas there were no such changes amongst AA and AG patients. For non-infarct fibrosis, patients who received O-3FA experienced a mean ECV regression of 1.7%, compared to 0.3% expansion in those treated with placebo in the overall cohort (p = 0.02). When stratified by genotype, AA and GG patients did not experience significant improvement in non-infarct fibrosis compared with placebo, whereas improvement in non-infarct myocardial fibrosis was noted in AG patients (change in non-infarct ECV -1.02±5.6 vs 3.1±7.8%, O-3FA vs placebo, p = 0.02). Reduction of infarct size, which occurred in both patients treated with O-3FA and placebo, was not statistically significant across the genotype groups (p = 0.32). There was a trend towards improvement of LVEF in patients treated with O-3FA versus those on placebo (p = 0.07), but the results were not statistically significant within each of the 3 genotype subgroups.

**Table 3:** Effectiveness of O-3FA in improving CMR markers of post-MI cardiac remodeling, stratified by FADS2 genotypes. All changes represent absolute changes from baseline to 6-month CMR.

	AA (N = 156)			AG (N = 122)			GG (N = 34)		
	Drug	Placebo	p-value	Drug	Placebo	p-value	Drug	Placebo	p-value
Change in LVESVi (ml/m^2^)	-2.5 ± 6.7	-1.0 ± 8.3	0.31	-3.6 ± 6.6	-1.2 ± 7.8	0.14	-4.4 ± 5.6	1.2 ± 4.1	**0.006**
Odds of ≥ 10% LVESVi Improvement	0.58	0.50	OR = 1.2	0.80	0.50	OR = 1.6	0.60	0.083	**OR = 7.2**
Change in LVEDVi (ml/m^2^)	-1.2 ± 12.4	-0.2 ± 13.2	0.69	-2.9 ± 12.3	-0.6 ± 12.5	0.42	-4.2 ± 9.5	2.3 ± 8.3	0.06
Change in Non-infarct Myocardial Fibrosis (%)	-2.0± 5.2	-1.1 ± 4.2	0.43	-1.0 ± 5.6	3.1 ± 7.8	**0.02**	2.0 ± 5.3	-2.9 ± 2.0	0.64
Change in Infarct Size (g)	-3.4 ± 8.2	-1.3 ± 7.1	0.13	0.03 ± 5.4	-1.6 ± 6.4	0.21	-1.8 ± 9.7	-3.5 ± 10.2	0.16
Change in LVEF (%)	2.3 ± 5.8	1.1 ± 6.5	0.30	2.8 ± 4.9	1.1 ± 7.1	0.24	2.4 ± 4.6	0.02 ± 4.8	0.19

### O-3FA treatment and biomarkers of cardiac remodeling and inflammation

**Fig 4:**
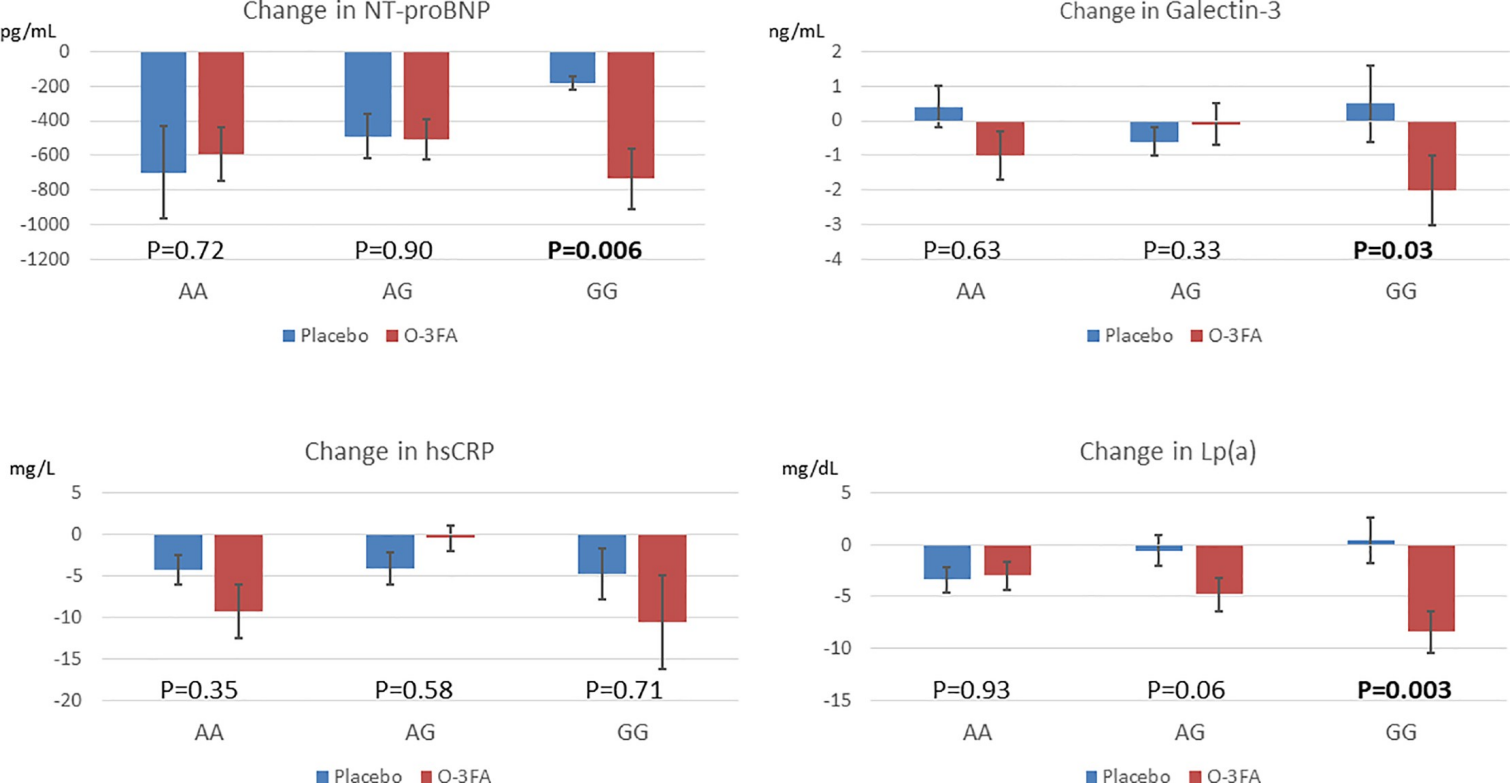
O-3FA treatment effects on cardiac remodeling related biomarkers. Patients with the GG genotype experienced substantial reduction of NT-proBNP, and Galectin-3, and serum lipoprotein A, when assigned O-3FA.

**Fig 4** illustrates the key biomarkers of interest. In the overall cohort, patients who received O-3FA achieved similar magnitude of reduction of NT-proBNP as patients who received placebo (-582±975 vs -539±1312 pg/mL, p = 0.79). Reduction of NT-proBNP was also similar between O-3FA compared with placebo in patients with either AA or AG genotypes. However, patients with GG genotype treated with O-3FAs experienced significantly greater reduction of NT-proBNP levels as compared with placebo (-733±672 pg/mL vs -181±139 pg/mL, p = 0.006). A similar pattern of reduction was seen for galectin-3, a novel biomarker of myocardial strain. There was no significant difference in the magnitude of reduction of galectin-3 between O-3FA patients compared to placebo patients in the overall cohort (-0.84±4.73 vs 0.003±3.58 ng/mL, p = 0.14). Reduction of galectin-3 was also similar between the treatment groups in patients with either AA or AG genotypes. However, patients with GG genotype treated with O-3FA underwent significant reduction at 6 months compared to placebo (-1.96±4.16 vs 0.51±3.97 ng/mL, p = 0.03). O-3FA also significantly reduced lipoprotein A levels within the GG genotype patients (-8.4±7.62 vs 0.43±8.26 mg/dL, p = 0.003), with a trend towards reduction in AG, but not AA genotypes. There were no significant differences for reduction in high-sensitivity C-reactive protein for the entire cohort, as well as between different genotypes.

### Change in EPA, DHA, and O3I Level and LV remodeling across genotypes

**Fig 5:**
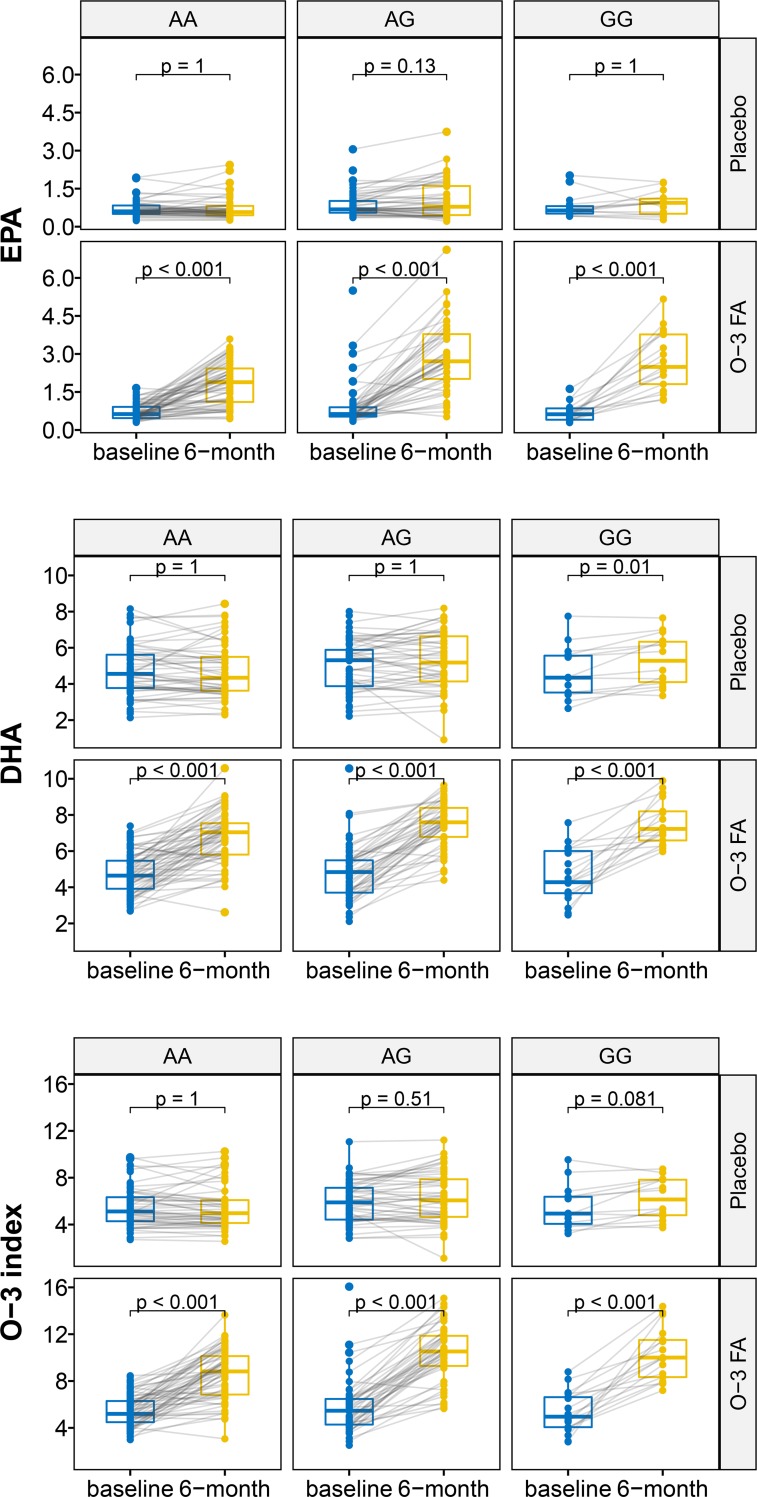
Effects on EPA, DHA, and O3I levels from treatment assignment, stratified by rs-1535 genotypes. P-values were adjusted by the Bonferroni method. The p-values came from paired Wilcoxon tests that compare for each sub-group the baseline against 6-month values.

**Fig 5** illustrates the baseline and 6-month levels of EPA, DHA, and O3I stratified by assigned treatment groups and rs1535 genotypes. Baseline levels of EPA, DHA, and O3I were not different between subgroups. Patients assigned to O-3 FA treatment across all 3 genotypes raised EPA, DHA, and O3I levels at 6 months, compared to placebo.

In the overall cohort, increases of EPA, DHA, and O3I from baseline to 6-months, demonstrated significant inverse correlations to change in LVESVi (Spearman correlation coefficients -0.17, -0.31, and -0.28; P-values 0.01, <0.0001, and <0.0001, respectively). **S1 Table** illustrates these correlations in the genotype subgroups. In patients with GG genotype, increases in EPA, DHA, and O3I were associated with reductions in LVESVi.

### Effects of race

Racial categories of the cohort included Caucasian (N = 251, 81%), Hispanic (N = 22, 7%), Black (N = 21, 7%), and other (N = 18, 5%). There was a trend towards higher proportion of Caucasian race with increasing number of G alleles. Therefore, additional analyses were performed to assess if the key study observations persisted in the Caucasian subgroup. The FADS2 activities by ArA/LA ratio of the Caucasian subgroup (N = 251, 81%) are shown in **S1 Fig**. Similar to the overall cohort, Caucasian patients with AA genotype had the highest ArA/LA ratio whereas those with GG genotype had the lowest (p<0.001), and AG patients had an intermediate level (AA vs AG, p<0.001).

Caucasian patients who received O-3FA experienced a trend towards improvement of LVESVi compared to those who received placebo (-1.02±7.49 vs -3.03±6.84 ml/m^2^, p = 0.06). Therapeutic effects of O-3FA treatment in Caucasian patients on cardiac remodeling and serum NT-proBNP, stratified by genotypes, are shown in **S2 Fig**. Caucasian patients with either AA or AG genotypes did not experience significant improvement of LVESVi from O-3FA treatment compared to placebo. However, Caucasian patients with GG genotype experienced a significant reduction of LVESVi when treated with O-3FA, compared with placebo (p = 0.01). A similar pattern was also seen in magnitude of reduction of NT-proBNP levels during the 6 months post-MI period, suggesting significant reduction of myocardial stretch when treating Caucasian GG-genotype patients with O-3FA (p = 0.006).

## Discussion

In this post-hoc analysis of the OMEGA-REMODEL trial, we evaluated for differential therapeutic responses from high-dose O-3FAs on post-AMI cardiac remodeling across polymorphisms of the FADS2 genotype. We observed that polymorphisms of the rs1535 gene directly correlated with FADS2 activity demonstrating an inverse linear trend for product to precursor ArA/LA ratios for those heterozygous and homozygous for G alleles. While there was a modest improvement in LVESVi in the overall study cohort, this therapeutic response was seen to be more robust in patients with the GG genotype. Interestingly, the improvement in cardiac remodeling for the GG genotype patients was paralleled by reduction in circulating biomarkers of myocardial strain (galectin-3) and stretch (NT-proBNP). These findings suggest that similar to observations in other inflammatory disease states[3], genetic profiling using rs1535 may differentiate patients’ response to O-3FA treatment and may lead to improved patient selection for O-3FA treatment in post-AMI remodeling.

Despite the decline in post-MI mortality owing to the successes of rapid emergent revascularization strategies and improved goal-directed medical therapies, the incidence of post-MI heart failure remains unacceptably high [17, 18]. Adverse cardiac remodeling, the key mechanistic link to post-AMI heart failure and mortality, is poorly predicted by baseline LVEF and infarct size measurements [7]. It has long been recognized that excessive and persistent activation of inflammatory pathways during infarct healing directly contributes to progressive adverse cardiac remodeling and is an important therapeutic target for prevention [7]. While myocardial inflammation is clearly implicated in adverse cardiac remodeling post-MI, all large-scale clinical trials to date that have focused on single-target anti-inflammatory therapies have been disappointing. These studies included evaluation of glucocorticoids, NSAIDs, in addition to specific antibodies against neutrophil adhesion molecules, complement cascade activation, and inflammatory cytokines [19]. Although speculative, one possible explanation may be that due to the inherent redundancy in inflammatory pathways activated post-MI, a single agent may be less efficacious than a strategy which regulates multiple pathways [7].

The anti-inflammatory effects of O-3FA are well established in both cardiovascular and non-cardiovascular systems. O-3FAs lower levels of key inflammatory eicosanoids, such as leukotriene-B4, thromboxane-A2, tumor necrosis factor (TNF), IL-1β, and IL-6 [20–23]. More recently, O-3FA has been demonstrated to serve as precursors to specialized pro-resolving lipid mediators (SPMs), that mediate resolution of the acute inflammatory response to myocardial infarction [24]. SPMs have shown to actively switch off leukocyte trafficking to inflamed sites, promote clearance of cellular debris and inflammatory mediators, and suppress cytokine production [25]. The ability of SPMs to modulate damage and subsequent fibroblast proliferation may be particularly important in models of ischemia and reperfusion, such as the convalescent phase following AMI [25]. Keyes et al, reported that resolvin E1 (RvE1) derived from EPA had direct protective effects on cardiomyocytes in a mouse model of ischemia/reperfusion. RvE1 reduced infarct size and apoptosis in a dose-dependent fashion [26]. Another SPM biosynthesized in vivo from EPA, resolvin D1 (RvD1), was evaluated in a similar mouse model of ischemia/reperfusion by Kain and colleagues [27]. They reported that the RvD1 injected group had early exit of neutrophils from both the LV and the spleen and switching to anti-inflammatory M2 macrophages with resultant decreased cardiac collagen deposition and improved LV function [27]. Furthermore, O-3FAs are oxidized by CYP450 monooxygenase to epoxides, which are potent lipid mediators of the cardiovascular system [28]. Epoxides have been show to induce vasodilation, stimulate angiogenesis and further protect the myocardial from ischemic/reperfusion injury [29, 30]. Most recently, Endo and colleagues reported that 18-HEPE, an anti-fibrotic and anti-inflammatory EPA metabolite, prevented cardiac dysfunction, macrophage infiltration, and cardiac fibrosis in a mouse model of cardiac pressure overload [31]. In the OMEGA-REMODEL trial, treatment with O-3FA treatment was associated with significant reduction of circulating biomarkers of inflammation, such as myeloperoxidase, and lipoprotein-associated phospholipase A2, which paralleled the improvement in cardiac remodeling [2]. In this post-hoc analysis, the GG genotype patients did not demonstrate a significant reduction in hs-CRP. There are several possible reasons serum hs-CRP may not capture the full spectrum of anti-inflammatory and remodeling effects from O-3FA across the genotypes. Local anti-inflammatory and proresolving effects of the SPMs in the healing infarct are not reflected by systemic hs-CRP. Given the small average infarct size and preserved LVEF within our study cohort, patients likely underwent a mild systemic inflammatory response post-MI and therefore our study is inadequately powered to detect a significant change in peripheral hs-CRP amongst the genotypes. Furthermore, by our study protocol, the study drug (O-3FA or placebo) was administered to patient subjects at 2–4 weeks after acute MI when the systemic inflammatory response measured by hs-CRP may have diminished.

In the past decade, genome-wide association studies have identified SNP variants associated with elevated or low blood levels of omega-3 fatty acids. For instance, variants in the fatty acid desaturase genes FADS1 and FADS2, involved in the production of EPA and DHA from α-linolenic acid, represent a source of genetic variation in circulating levels of O-3FA, especially in individuals who consume very little to no dietary O-3FAs [32, 33]. Currently, at least a dozen genetic loci have been shown to affect circulating levels of O-3FA, many with still unclear mechanisms of action [34]. A few studies have directly examined the effects of FADS genotype on desaturase activity and coronary artery disease. In the Verona Heart Study, 610 patients with and 266 patients without CAD underwent genotyping for FADS polymorphisms. Four variants associated with elevated ArA/LA ratio were also associated with higher hs-CRP and greater risk of CAD [5].

SNP variation at rs1535 is of particular interest for two reasons: 1) it has minor allele frequency of sufficient prevalence (0.32) to be of relevance in standard clinical practice, and 2) prior studies have demonstrated effect modification of rs1535 genotype on impact of increasing O-3FA dietary intake [3, 4]. Studies on the impact of breastfeeding on child IQ, for instance, have suggested that the effect may be most pronounced in those who carry one copy of the A allele [4]. Bisgaard and colleagues examined the effect of maternal omega-3 fatty acid supplementation on the risk of asthma and wheezing in the offspring, which may result from a proinflammatory state due to imbalance between O-3FA and O-6FA levels in the infants [3]. They conducted a randomized, placebo-controlled trial of moderate-dose O-3FAs (2.4g/day) in 736 pregnant women initiated at week 24 of pregnancy. In the offsprings, risk of persistent wheeze or asthma was 30.7% lower in those with maternal O-3FA therapy. Notably, post hoc analyses showed that the G allele was associated with higher levels of upstream substrates (linoleic acid and alpha-linolenic acid) and predicted those most likely to benefit from therapy (GG genotype). We found similar results for high-dose O-3FA therapy in this post-hoc analysis of the OMEGA-REMODEL study.

There are several limitations to this study. First, we were not able to genotype 46 of the original 358 patients (13%, 20 assigned to O3-FA) in our cohort due to either patient or technical issues. Although this 13% was evenly distributed in both treatment arms, biases could potentially have been introduced. Second, our sample size was comparatively small and may have limited our power to fully evaluate the relationship of fatty acid increases with changes in LVESVi in the genotype groups, particularly in homozygote GG patients. Third, our study did not assess all available biomarkers that examines the inflammatory and proresolving effects of O-3FA and was inadequately powered to assess systemic inflammatory response by hs-CRP across the genotypes. Finally, our population was predominantly Caucasian, which limited the evaluation for any modifying effects due to race/ethnicity in response to O-3FA therapy/FADS2 genotypic polymorphisms. Nevertheless, our results support the concept that polymorphisms affecting FADS2 enzyme activity may modify the effect of O-3FA supplementation in Caucasian populations.

## Conclusions

In conclusion, our study demonstrated that the therapeutic effects of high dose O-3FA treatment in patients with acute MI was modified by polymorphisms of FADS2 gene at rs1535. Measures of improved LV remodeling, namely lower LVESVi and reduced non-infarct myocardial fibrosis, were greatest in patients with the homozygous GG genotype. Our findings support the concept of ‘precision medicine’ and that genotypic patient selection for O-3FA therapy post-AMI may yield improved clinical outcomes.

## Supporting information

S1 Fig Fatty acid desaturase activity in caucasian patients. Fatty acid desaturase activity as assessed by ArA/LA ratio in red blood cell membrane assay. Caucasian patients with GG alleles have diminished ArA/LA ratio consistent with reduced fatty acid desaturase activity.
(TIF)

S2 Fig Treatment assignment and reduction of adverse LV remodeling and NTpro-BNP amongst caucasian patients. (TIF)

S1 Table Correlations of changes in LVESVi with changes in EPA, DHA, and O3I stratified by genotype subgroups. (DOCX)

S1 Dataset Source dataset of all data analyses. (XLSX)

## References

[1] Bhatt AS, Ambrosy AP, Velazquez EJ. Adverse Remodeling and Reverse Remodeling After Myocardial Infarction. Curr Cardiol Rep. 2017;19(8):71 Epub 2017/07/01. 10.1007/s11886-017-0876-4 . 28660552

[2] Heydari B, Abdullah S, Pottala JV, Shah R, Abbasi S, Mandry D, et al Effect of Omega-3 Acid Ethyl Esters on Left Ventricular Remodeling After Acute Myocardial Infarction: The OMEGA-REMODEL Randomized Clinical Trial. Circulation. 2016;134(5):378–91. Epub 2016/08/03. 10.1161/CIRCULATIONAHA.115.019949 27482002 PMC4973577

[3] Bisgaard H, Stokholm J, Chawes BL, Vissing NH, Bjarnadottir E, Schoos AM, et al Fish Oil-Derived Fatty Acids in Pregnancy and Wheeze and Asthma in Offspring. N Engl J Med. 2016;375(26):2530–9. Epub 2016/12/29. 10.1056/NEJMoa1503734 . 28029926

[4] Caspi A, Williams B, Kim-Cohen J, Craig IW, Milne BJ, Poulton R, et al Moderation of breastfeeding effects on the IQ by genetic variation in fatty acid metabolism. Proc Natl Acad Sci U S A. 2007;104(47):18860–5. Epub 2007/11/07. 10.1073/pnas.0704292104 17984066 PMC2141867

[5] Martinelli N, Girelli D, Malerba G, Guarini P, Illig T, Trabetti E, et al FADS genotypes and desaturase activity estimated by the ratio of arachidonic acid to linoleic acid are associated with inflammation and coronary artery disease. Am J Clin Nutr. 2008;88(4):941–9. Epub 2008/10/10. 10.1093/ajcn/88.4.941 . 18842780

[6] Jiang B, Liao R. The paradoxical role of inflammation in cardiac repair and regeneration. J Cardiovasc Transl Res. 2010;3(4):410–6. 10.1007/s12265-010-9193-7 . 20559773

[7] Westman PC, Lipinski MJ, Luger D, Waksman R, Bonow RO, Wu E, et al Inflammation as a Driver of Adverse Left Ventricular Remodeling After Acute Myocardial Infarction. Journal of the American College of Cardiology. 2016;67(17):2050–60. Epub 2016/04/30. 10.1016/j.jacc.2016.01.073 . 27126533

[8] Sansbury BE, Spite M. Resolution of Acute Inflammation and the Role of Resolvins in Immunity, Thrombosis, and Vascular Biology. Circ Res. 2016;119(1):113–30. Epub 2016/06/25. 10.1161/CIRCRESAHA.116.307308 27340271 PMC5260827

[9] Serhan CN, Clish CB, Brannon J, Colgan SP, Chiang N, Gronert K. Novel functional sets of lipid-derived mediators with antiinflammatory actions generated from omega-3 fatty acids via cyclooxygenase 2-nonsteroidal antiinflammatory drugs and transcellular processing. J Exp Med. 2000;192(8):1197–204. Epub 2000/10/18. 10.1084/jem.192.8.1197 11034610 PMC2195872

[10] Serhan CN, Hong S, Gronert K, Colgan SP, Devchand PR, Mirick G, et al Resolvins: a family of bioactive products of omega-3 fatty acid transformation circuits initiated by aspirin treatment that counter proinflammation signals. J Exp Med. 2002;196(8):1025–37. Epub 2002/10/23. 10.1084/jem.20020760 12391014 PMC2194036

[11] Leaf A, Weber PC. A new era for science in nutrition. Am J Clin Nutr. 1987;45(5 Suppl):1048–53. Epub 1987/05/01. 10.1093/ajcn/45.5.1048 . 3578100

[12] Kim RJ, Wu E, Rafael A, Chen EL, Parker MA, Simonetti O, et al The use of contrast-enhanced magnetic resonance imaging to identify reversible myocardial dysfunction. The New England journal of medicine. 2000;343(20):1445–53. Epub 2000/11/18. 10.1056/NEJM200011163432003 . 11078769

[13] Cerqueira MD, Weissman NJ, Dilsizian V, Jacobs AK, Kaul S, Laskey WK, et al Standardized myocardial segmentation and nomenclature for tomographic imaging of the heart. A statement for healthcare professionals from the Cardiac Imaging Committee of the Council on Clinical Cardiology of the American Heart Association. Circulation. 2002;105(4):539–42. Epub 2002/01/30. 10.1161/hc0402.102975 . 11815441

[14] Deichmann R HA. Quantification of T1 Values by SNAPSHOT-FLASH NMR imaging. Journal of Magnetic Resonance. 1992;96:608–12.

[15] Coelho-Filho OR, Mongeon FP, Mitchell R, Moreno H Jr., Nadruz W Jr., Kwong R, et al Role of transcytolemmal water-exchange in magnetic resonance measurements of diffuse myocardial fibrosis in hypertensive heart disease. Circulation Cardiovascular imaging. 2013;6(1):134–41. Epub 2012/11/20. 10.1161/CIRCIMAGING.112.979815 23159497 PMC3587170

[16] Jerosch-Herold M, Sheridan DC, Kushner JD, Nauman D, Burgess D, Dutton D, et al Cardiac magnetic resonance imaging of myocardial contrast uptake and blood flow in patients affected with idiopathic or familial dilated cardiomyopathy. American journal of physiology Heart and circulatory physiology. 2008;295(3):H1234–H42. Epub 2008/07/29. 10.1152/ajpheart.00429.2008 18660445 PMC2544489

[17] Desta L, Jernberg T, Lofman I, Hofman-Bang C, Hagerman I, Spaak J, et al Incidence, temporal trends, and prognostic impact of heart failure complicating acute myocardial infarction. The SWEDEHEART Registry (Swedish Web-System for Enhancement and Development of Evidence-Based Care in Heart Disease Evaluated According to Recommended Therapies): a study of 199,851 patients admitted with index acute myocardial infarctions, 1996 to 2008. JACC Heart Fail. 2015;3(3):234–42. Epub 2015/03/07. 10.1016/j.jchf.2014.10.007 . 25742760

[18] Hung J, Teng TH, Finn J, Knuiman M, Briffa T, Stewart S, et al Trends from 1996 to 2007 in incidence and mortality outcomes of heart failure after acute myocardial infarction: a population-based study of 20,812 patients with first acute myocardial infarction in Western Australia. J Am Heart Assoc. 2013;2(5):e000172 Epub 2013/10/10. 10.1161/JAHA.113.000172 24103569 PMC3835218

[19] Seropian IM, Toldo S, Van Tassell BW, Abbate A. Anti-inflammatory strategies for ventricular remodeling following ST-segment elevation acute myocardial infarction. Journal of the American College of Cardiology. 2014;63(16):1593–603. Epub 2014/02/18. 10.1016/j.jacc.2014.01.014 . 24530674

[20] Mozaffarian D, Wu JH. Omega-3 fatty acids and cardiovascular disease: effects on risk factors, molecular pathways, and clinical events. Journal of the American College of Cardiology. 2011;58(20):2047–67. Epub 2011/11/05. 10.1016/j.jacc.2011.06.063 . 22051327

[21] Gani OA, Sylte I. Molecular recognition of docosahexaenoic acid by peroxisome proliferator-activated receptors and retinoid-X receptor alpha. J Mol Graph Model. 2008;27(2):217–24. Epub 2008/06/13. 10.1016/j.jmgm.2008.04.008 . 18547851

[22] Oh DY, Talukdar S, Bae EJ, Imamura T, Morinaga H, Fan W, et al GPR120 is an omega-3 fatty acid receptor mediating potent anti-inflammatory and insulin-sensitizing effects. Cell. 2010;142(5):687–98. Epub 2010/09/04. 10.1016/j.cell.2010.07.041 20813258 PMC2956412

[23] Zhao Y, Joshi-Barve S, Barve S, Chen LH. Eicosapentaenoic acid prevents LPS-induced TNF-alpha expression by preventing NF-kappaB activation. J Am Coll Nutr. 2004;23(1):71–8. Epub 2004/02/14. 10.1080/07315724.2004.10719345 . 14963056

[24] Spite M, Claria J, Serhan CN. Resolvins, specialized proresolving lipid mediators, and their potential roles in metabolic diseases. Cell Metab. 2014;19(1):21–36. Epub 2013/11/19. 10.1016/j.cmet.2013.10.006 24239568 PMC3947989

[25] Serhan CN. Pro-resolving lipid mediators are leads for resolution physiology. Nature. 2014;510(7503):92–101. Epub 2014/06/06. 10.1038/nature13479 24899309 PMC4263681

[26] Keyes KT, Ye Y, Lin Y, Zhang C, Perez-Polo JR, Gjorstrup P, et al Resolvin E1 protects the rat heart against reperfusion injury. Am J Physiol Heart Circ Physiol. 2010;299(1):H153–64. Epub 2010/05/04. 10.1152/ajpheart.01057.2009 . 20435846

[27] Kain V, Ingle KA, Colas RA, Dalli J, Prabhu SD, Serhan CN, et al Resolvin D1 activates the inflammation resolving response at splenic and ventricular site following myocardial infarction leading to improved ventricular function. J Mol Cell Cardiol. 2015;84:24–35. Epub 2015/04/15. 10.1016/j.yjmcc.2015.04.003 25870158 PMC4468047

[28] Endo J, Arita M. Cardioprotective mechanism of omega-3 polyunsaturated fatty acids. J Cardiol. 2016;67(1):22–7. Epub 2015/09/12. 10.1016/j.jjcc.2015.08.002 . 26359712

[29] Liu Y, Wang R, Li J, Rao J, Li W, Falck JR, et al Stable EET urea agonist and soluble epoxide hydrolase inhibitor regulate rat pulmonary arteries through TRPCs. Hypertens Res. 2011;34(5):630–9. Epub 2011/02/11. 10.1038/hr.2011.5 21307870 PMC4548928

[30] Spector AA, Kim HY. Cytochrome P450 epoxygenase pathway of polyunsaturated fatty acid metabolism. Biochim Biophys Acta. 2015;1851(4):356–65. Epub 2014/08/06. 10.1016/j.bbalip.2014.07.020 25093613 PMC4314516

[31] Endo J, Sano M, Isobe Y, Fukuda K, Kang JX, Arai H, et al 18-HEPE, an n-3 fatty acid metabolite released by macrophages, prevents pressure overload-induced maladaptive cardiac remodeling. J Exp Med. 2014;211(8):1673–87. Epub 2014/07/23. 10.1084/jem.20132011 25049337 PMC4113943

[32] Lemaitre RN, Tanaka T, Tang W, Manichaikul A, Foy M, Kabagambe EK, et al Genetic loci associated with plasma phospholipid n-3 fatty acids: a meta-analysis of genome-wide association studies from the CHARGE Consortium. PLoS Genet. 2011;7(7):e1002193 Epub 2011/08/11. 10.1371/journal.pgen.1002193 21829377 PMC3145614

[33] Tanaka T, Shen J, Abecasis GR, Kisialiou A, Ordovas JM, Guralnik JM, et al Genome-wide association study of plasma polyunsaturated fatty acids in the InCHIANTI Study. PLoS Genet. 2009;5(1):e1000338 Epub 2009/01/17. 10.1371/journal.pgen.1000338 19148276 PMC2613033

[34] Hu Y, Li H, Lu L, Manichaikul A, Zhu J, Chen YD, et al Genome-wide meta-analyses identify novel loci associated with n-3 and n-6 polyunsaturated fatty acid levels in Chinese and European-ancestry populations. Hum Mol Genet. 2016;25(6):1215–24. Epub 2016/01/09. 10.1093/hmg/ddw002 26744325 PMC4764197

