# Supplementary figures and images for: Genetic profiling of fatty acid desaturase polymorphisms identifies patients who may benefit from high-dose omega-3 fatty acids in cardiac remodeling after acute myocardial infarction—Post-hoc analysis from the OMEGA-REMODEL randomized controlled trial
Source: PLoS One. 2019 Sep 18;14(9):e0222061. doi: 10.1371/journal.pone.0222061 (PMC6750606; doi:10.1371/journal.pone.0222061)

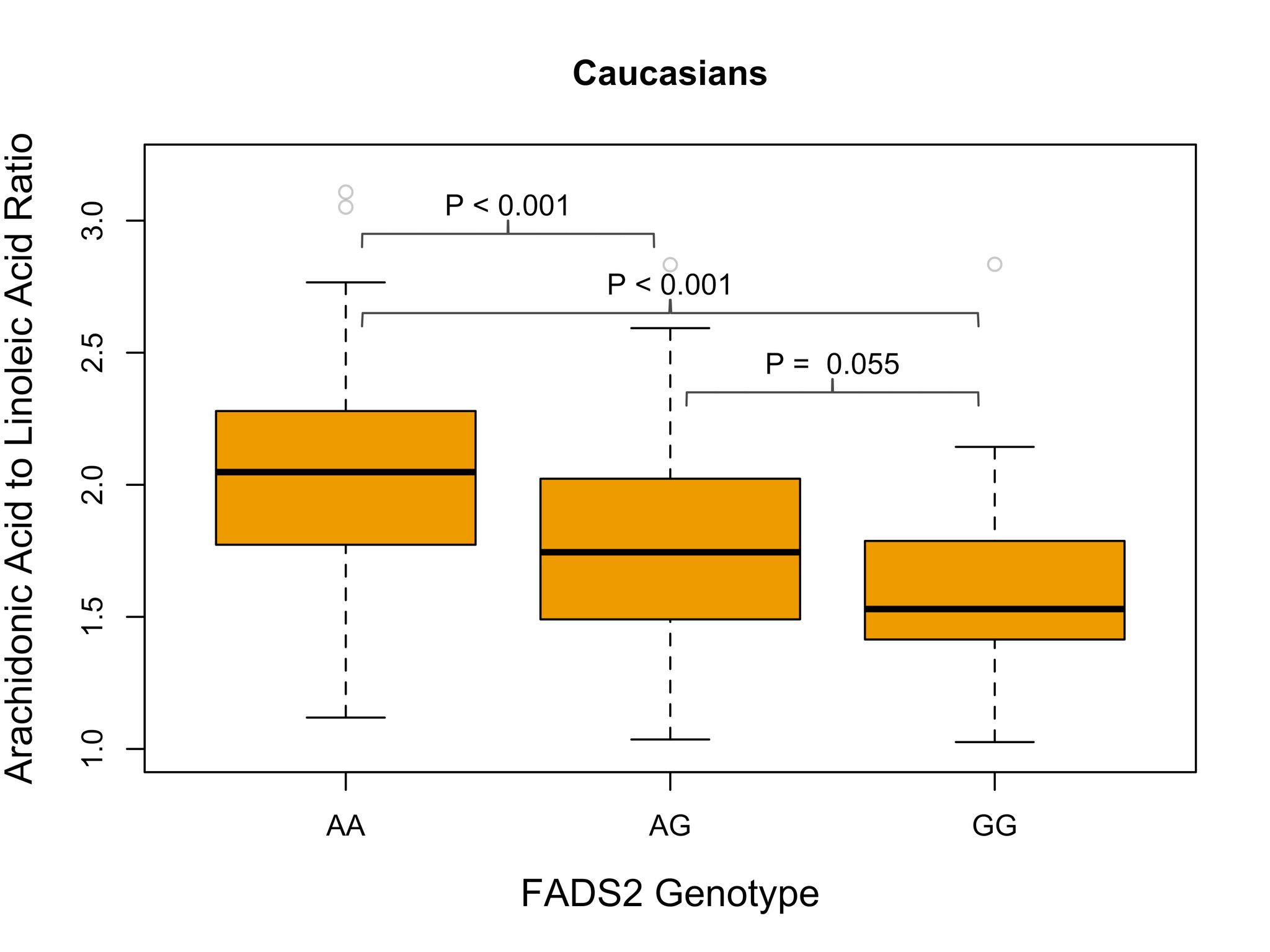

Supplement: S1 Fig — Fatty acid desaturase activity as assessed by ArA/LA ratio in red blood cell membrane assay. Caucasian patients with GG alleles have diminished ArA/LA ratio consistent with reduced fatty acid desaturase activity. (TIF) [file pone.0222061.s001.TIF]

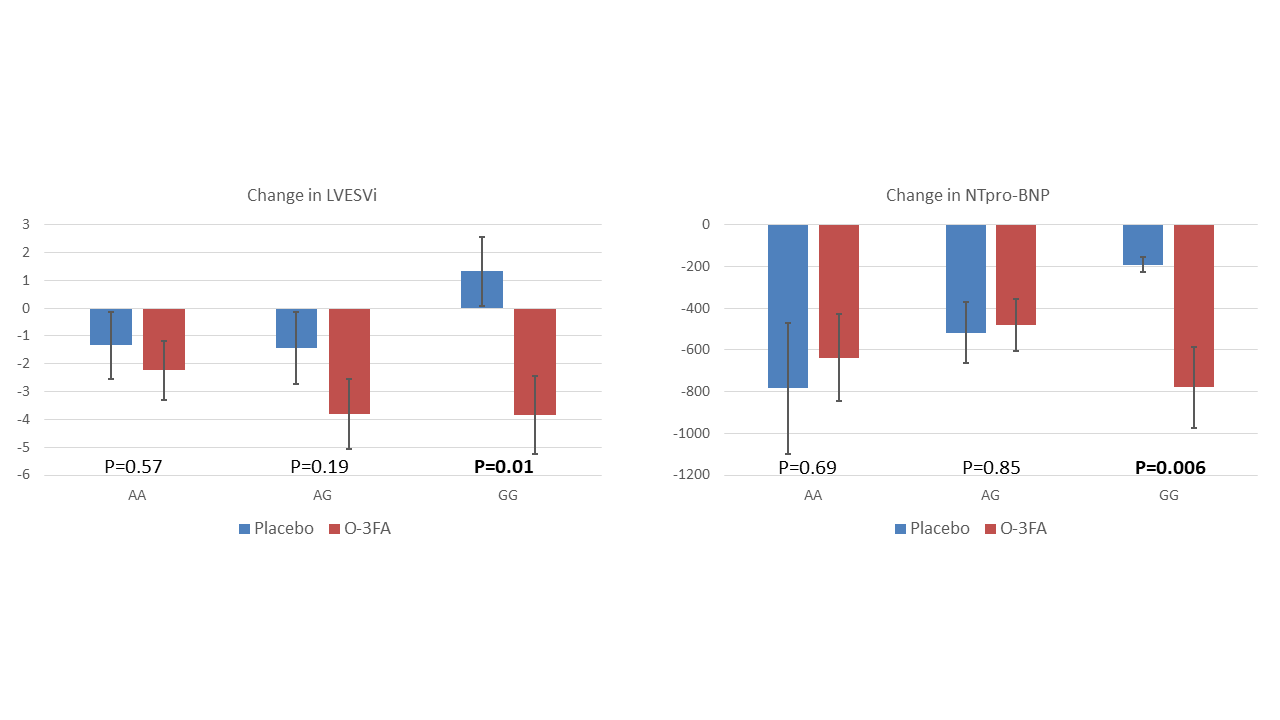

Supplement: S2 Fig — (TIF) [file pone.0222061.s002.TIF]
