# Supplementary material for: Genetic profiling of fatty acid desaturase polymorphisms identifies patients who may benefit from high-dose omega-3 fatty acids in cardiac remodeling after acute myocardial infarction—Post-hoc analysis from the OMEGA-REMODEL randomized controlled trial
Source: PLoS One. 2019 Sep 18;14(9):e0222061. doi: 10.1371/journal.pone.0222061 (PMC6750606; doi:10.1371/journal.pone.0222061)
Supplement: S1 Table — (DOCX) [file pone.0222061.s003.docx]

**Supplemental Table 1**

|  | | **EPA** | **DHA** | **O3I** |
| --- | --- | --- | --- | --- |
| **Change in LVESVi from baseline** | **Whole Cohort** | -0.17, p=0.01 | -0.31, **p<0.0001** | -0.28, **p<0.0001** |
|  | **AA genotype** | -0.14, p=0.15 | -0.29, **p=0.003** | -0.26, **p=0.008** |
|  | **AG genotype** | -0.13, p=0.27 | -0.32, **p=0.005** | -0.27, **p=0.02** |
|  | **GG genotype** | -0.43, **p=0.02** | -0.40, **p=0.03** | -0.46, **p=0.01** |
